# The Loneliness Epidemic: Unveiling its Impact on Odontogenic Infections. A Retrospective Cohort Study

**DOI:** 10.3290/j.ohpd.c_2382

**Published:** 2025-12-02

**Authors:** Jussi Furuholm, Aleksi Haapanen, Johanna Snäll

**Affiliations:** a Jussi Furuholm Researcher, Department of Oral and Maxillofacial Diseases, University of Helsinki, Finland. performed statistical analysis and prepared the manuscript, involved in revising the manuscript, provided intellectual input, read and approved the final manuscript.; b Aleksi Haapanen Researcher, Department of Oral and Maxillofacial Diseases, University of Helsinki, Finland. Drafted the manuscript and developed the study design, involved in revising the manuscript, provided intellectual input, read and approvied the final manuscript.; c Johanna Snäll Associate Professor, Department of Oral and Maxillofacial Diseases, University of Helsinki, Finland. Drafted the manuscript and developed the study design, scrutinised the patient records and collected the raw data, involved in revising the manuscript, provided intellectual input, read and approved the final manuscript.

**Keywords:** dental, infection control, loneliness, oral health, social class.

## Abstract

**Purpose:**

Socioeconomic conditions and loneliness are emerging as important determinants of oral and general health. This study explores their impact on odontogenic infections (OIs) severe enough to require hospitalization.

**Materials and Methods:**

This retrospective cohort study utilized data of patients with emergency visits to the oral and maxillofacial emergency unit from January 2012 to October 2020 at Helsinki University Hospital. The data were combined with Statistics Finland to analyze the association between socioeconomic factors and OIs. Bivariate comparisons and binomial logistic regression analysis were used to investigate the effect of selected sociodemographic and socioeconomic factors on OIs requiring hospitalization.

**Results:**

The study included 2838 patients, of which 709 (25.0%) were hospitalized. Patients with OIs resulting in hospitalization were statistically significantly more often single, divorced, or widowed, than married (p<0.001). Additionally, rental living (p=0.049), a lower level of education (p=0.018), and lower income level (p=0.002) were associated with hospitalization. After adjusting for age, sex, and living arrangement, a marital status of single, divorced, or widowed (odds ratio, OR=1.406, 95% CI 1.148–1.723, p=0.001) and lowest income level (ref. highest income level, OR=1.418, 95% CI 1.090–1.845, p=0.009) predicted hospitalization in logistic regression analysis.

**Conclusion:**

OIs resulted more often in hospitalization if the patient was single, divorced, or widowed, or had a lower income level. These findings highlight the importance of implementing comprehensive public health strategies that tackle socioeconomic disparities to enhance oral health outcomes and reduce the impact of severe dental infections.

Various socioeconomic factors may have an impact on oral health conditions.^1,4,14,18,21^ One of the most critical socioeconomic factors is access to dental care.^22^ People from lower socioeconomic backgrounds often have less access to dental services, which can lead to delays in the diagnosis and treatment of dental infections. This lack of access can be due to financial constraints, lack of insurance, geographic barriers, or insufficient dental health literacy. The mean travel distance to dental care has been found to be greater for patients with more severe markers of odontogenic infections (OIs) than for patients with minor infections.^20^

The World Health Organisation (WHO) launched a Commission on Social Connection in 2023 to focus on recognizing loneliness and isolation as a global public health concern.^26^ Loneliness and social isolation are considered a major risk for premature death, and they increase the risk for multiple diseases.^3,10,23^ This increased health risk can be quantified as smoking 15 cigarettes daily.^9^ Objective social isolation—rather than subjective loneliness—has also been shown to increase healthcare expenditures.^19^

An association has been observed between higher caries index scores and lower educational levels.^16^ Higher levels of education often correlate with better oral health practices and greater awareness of the importance of seeking dental care; conversely, individuals with lower levels of education might lack knowledge about proper dental hygiene practices and the significance of regular dental check-ups.^17^ Education level may also play a significant role in the prevalence and management of OIs. Irregular dental care has been shown to increase the risk for oral infections with the potential for spread.^6^ Caries and apical periodontitis are major causes of OIs, underscoring the critical role of effective primary dental care in their prevention.^2,7^

Marital status has been observed to affect accessibility to dental care in men: non-married men were more unlikely to receive dental treatment than married men.^12^ In a Chinese study, a higher living standard was found to be associated with more frequent utilization of dental care.^13^ On a more general level, sociodemographic changes and their influence on oral health disparities and oral healthcare needs have been discussed in a recent study by Inglehart et al.^11^

We designed and conducted a retrospective cohort study to examine the effects of socioeconomic factors on OIs requiring hospitalization. Our study hypothesis was that lower socioeconomic levels are associated with more severe OIs.

## MATERIALS AND METHODS

### Ethics Approval

The study protocol was approved by the Internal Review Board of the Head and Neck Center, Helsinki University Hospital, Helsinki, Finland (HUS/148/2022). Additionally, permission was granted by Statistics Finland (TK/1917/07.03.00/2022). The study was conducted in adherence to the Declaration of Helsinki.

This study is reported according to STROBE (Strengthening the Reporting of Observational Studies in Epidemiology) guidelines.^24^ The study is part of the Sociodemographic Factors Behind Craniomaxillofacial Emergencies (SOCE) Trial.

Data of all patients with acute infections evaluated in the oral and maxillofacial emergency unit in Department of Oral and Maxillofacial Surgery, Helsinki University Hospital, Helsinki, Finland, between January 2012 and October 2020 were retrospectively reviewed. The emergency unit is the largest oral and maxillofacial on-call hospital unit in Finland, with a catchment area of 1.6 million inhabitants. The patient data were retrieved from the hospital’s electronic patient database. Patients were selected based on emergency visit International Statistical Classification of Diseases and Related Health Problems (ICD) diagnosis codes.

The patient records were combined with the FOLK personal data module from Statistics Finland (https://aineistokatalogi.fi/catalog/studies/a6946178-3c4d-432e-b4bd-7b32b80932af). The FOLK data included information on the following parameters: socioeconomic factors, income level, family relationships, housing quality, living environment, marital status, and country of birth.

Patients evaluated in the oral and maxillofacial surgery department for a suspected or acute infection were included in the present study. Excluded were patients without available data in the FOLK personal data module because the patient did not have a Finnish personal identification number or they were already deceased before the time of data extraction.

**Fig 1:**
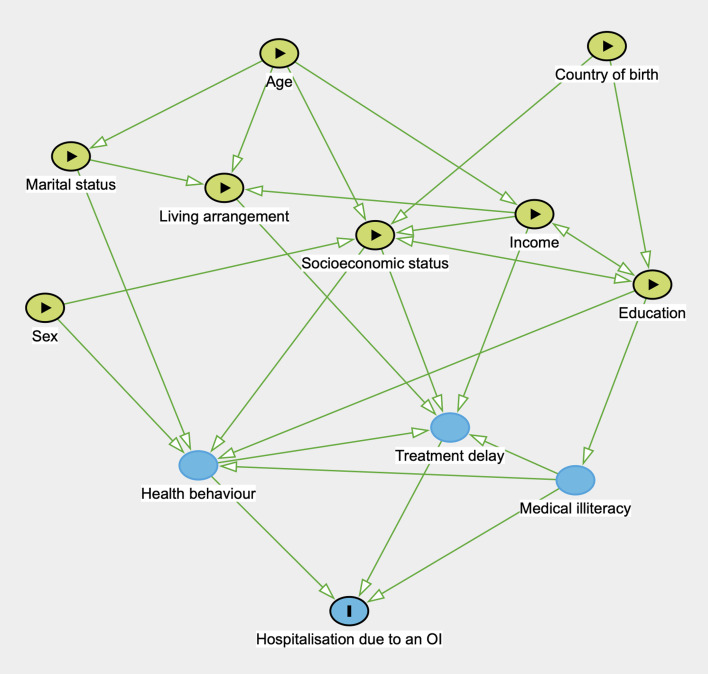
A directed acyclic graph describing the relationships among explanatory, predictor, and outcome variables. OI=odontogenic infection.

The main outcome variable was hospitalization. The predictor variables were country of birth, marital status, education level, living arrangement, employment status, and income level. The explanatory variables were age and sex. Explanatory and predictor variables were compared between hospitalized and non-hospitalized OI patients. Figure 1 illustrates the relationships among the outcome, predictor, and explanatory variables.

All analyses were performed with SPSS for Macintosh, version 28.0 (IBM; Armonk, NY, USA). Descriptive statistics are presented as frequencies with proportions or means with standard deviations. Categorical variables were cross-tabulated and analyzed with Pearson’s chi-squared test. In continuous variables, Student’s t-test and Mann–Whitney U-test were used to compare differences between study groups. As post-hoc analyses, pairwise comparisons were performed for Pearson’s chi-squared test using the Z-test and Dunn’s (1964) procedure for the Kruskal–Wallis H-test, both with a Bonferroni correction for multiple comparisons. A binomial logistic regression model was created after bivariate analyses. Explanatory and statistically significant predictor variables were selected in the model; education level was omitted due to missing values and multicollinearity. Odds ratios (OR) with 95% confidence intervals (CI) were reported.

## RESULTS

**Fig 2:**
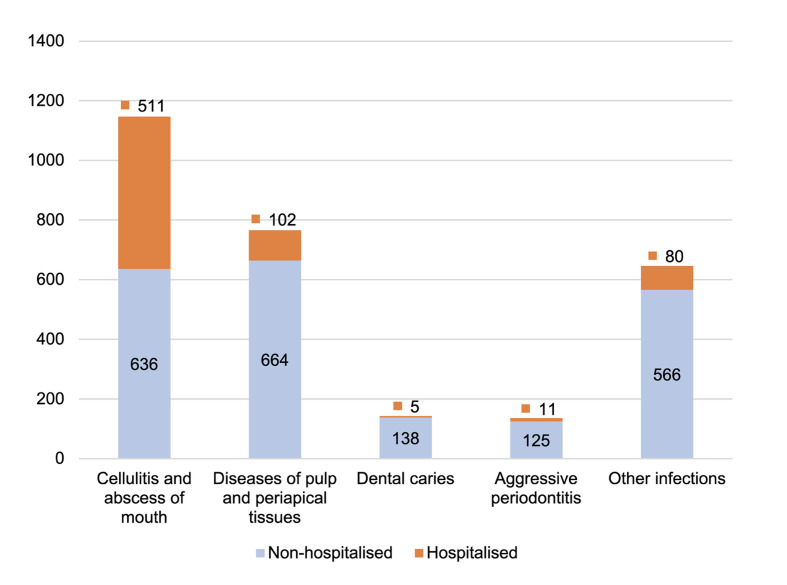
Distribution of infection diagnoses stratified by hospitalisation.

Altogether, 2838 patients’ records were included in the study; of those, 709 (25.0%) were hospitalized. The most common infection diagnoses were cellulitis and abscess of the mouth, and diseases of the pulp and periapical tissues (Fig 2).

The median age of all patients was 47 years, ranging from 0 to 100 years, and 54.0% of the patients were male (Table 1). Hospitalized patients were statistically significantly more often single, divorced, or widowed, than married (p<0.001). In addition, rental living (p=0.049), a lower level of education (p=0.018), and lower income level (p=0.002) were associated with hospitalization. In pairwise comparisons, no statistically significant differences were observed in patients’ age, sex, country of birth, or employment status.

**Table 1:** Patient demographics and association of sociodemographic factors and hospitalisation due to infection

	Total (N = 2838)	Hospitalized (n = 709)	Non-hospitalized (n = 2129)	p-value	Effect size, if statistically significant
	n	valid % of n	n	valid % of n	n	valid % of n		
**Age (years)**
mean±SD	47.3±19.19	46.6±18.74	47.6±19.33	0.248	
median (min–max)	47 (0–100)	45 (10–100)	48 (0–96)	0.097	
≤47 (n, %)	1435, 50.6%	380, 53.6%	1055, 49.6%	0.062	
>47 (n, %)	1403, 49.4%	329, 46.4%	1074, 50.4%
**Sex**							0.133	
Male	1532	54.0	400	56.4	1132	53.2		
Female	1306	46.0	309	43.6	997	46.8		
**Country of birth**							0.630	
Finland	2532	89.2	636	89.7	1896	89.1		
Other	306	10.8	73	10.3	233	10.9		
**Marital status**							<0.001	0.084
Married	1007	35.5	202	28.5	805	37.8	*	
Single, divorced, or widowed	1831	64.5	507	71.5	1324	62.2	*	
**Education level (n=1775 with available data; n=1063 with missing data)**							0.018	0.056
Secondary	1035	58.3	270	63.2	765	56.8	*	
Tertiary or higher	740	41.7	157	36.8	583	43.2	*	
**Living arrangement (n=2689 with available data; n=149 with missing data)**							0.049	0.047
Own	1386	51.5	313	47.4	1073	52.9	*	
Rental	1166	43.4	309	46.8	857	42.2	*	
Other or unknown	137	5.1	38	5.8	99	4.9		
**Employment status**							0.712	
Employed	1378	48.6	340	48.0	1038	48.8		
Unemployed, student, pensioner, other	1460	51.4	369	52.0	1091	51.2		
**Income level (n=2757 with available data; n=81 with missing data)**							0.002	0.059
Lowest quartile	690	25.0	209	29.9	481	23.4	*	
2nd lowest quartile	704	25.5	179	25.6	525	25.5		
2nd highest quartile	679	24.6	164	23.5	515	25.0		
Highest quartile	684	24.8	146	20.9	538	26.1	*	
*Significant column difference with Z-test with Bonferroni correction.

Binomial logistic regression showed that marital status single, divorced, or widowed (ref. married, OR=1.406, 95% CI 1.148–1.723, p=0.001) and lowest income level (ref. highest income level, OR=1.418, 95% CI 1.090–1.845, p=0.009) statistically significantly predicted hospitalization after adjusting for age, sex, and living arrangement (Table 2).

**Table 2:** Logistic regression model predicting the likelihood of hospitalisation

	OR	95% CI	p-value
**Age, continuous**			
year	0.997	0.992–1.002	0.295
**Sex, ref. female**			
male	1.179	0.984–1.414	0.074
**Marital status, ref. married**			
single, divorced, or widowed	1.406	1.148–1.723	0.001
**Living arrangement, ref. own**			
rental	1.044	0.856–1.274	0.669
other or unknown	1.202	0.801–1.802	0.374
**Income level, ref. highest quartile**			
2nd highest quartile	1.146	0.883–1.487	0.304
2nd lowest quartile	1.166	0.894–1.520	0.257
lowest quartile	1.418	1.090–1.845	0.009
OR: odds ratio; CI: confidence interval.

## DISCUSSION

The present study offers insights into the influence of socioeconomic factors on the hospitalization rates of patients with OIs. Our study hypothesis was confirmed, as lower socioeconomic levels had a statistically significant effect on more severe OIs. By analyzing patient data from the largest maxillofacial emergency unit in Finland over eight years, we were able to identify critical socioeconomic determinants that affect the management and outcomes of these infections.

Interestingly, marital status emerged as a statistically significant predictor of hospitalization. Single, divorced, or widowed individuals had higher odds of being hospitalized compared to their married counterparts. This could be due to the social and emotional support that married individuals typically have, which might encourage better health practices and more timely healthcare-seeking behavior. Additionally, the financial stability often associated with marriage might enable better access to preventive dental care. These findings are in line with earlier observations linking loneliness and increased susceptibility to severe infections.^5^

Our findings underscore the importance of access to dental care as a critical socioeconomic factor. While the study did not directly measure access to dental services, the correlation between lower socioeconomic status and higher hospitalization rates suggests a lack of timely dental care. Patients from lower-income groups and those with lower levels of education—who likely have limited access to dental services due to financial constraints, lack of insurance, or insufficient health literacy—showed a higher propensity for hospitalization. This may be due to delay in accessing necessary dental care and lead to the progression of more severe infections. On the other hand, overall health and, for example, substance abuse status may also be poorer and predispose individuals to more severe infections. In addition, loneliness has been observed to be associated with postponed dental appointments and thus may lead to deteriorating oral health.^25^

Educational level also emerged as a statistically significant predictor in our study, consistent with the hypothesis that lower educational attainment correlates with poorer oral health outcomes. Individuals with lower levels of education might not only be less aware of the importance of regular dental check-ups but may also lack knowledge about proper dental hygiene practices.^15^ This lack of awareness can lead to the neglect of early symptoms of dental infections, resulting in severe conditions that require hospitalization. Although educational level had to be omitted from the logistic regression model due to multicollinearity, its statistically significant association with hospitalization in the bivariate analysis suggests a potential explanatory role.

Our study found that patients living in rental accommodations were more likely to be hospitalized, suggesting a link between poorer living conditions and more severe dental infections. Rental living might be indicative of lower socioeconomic status. This finding aligns with previous research indicating that suboptimal living conditions contribute to the spread and severity of infections, possibly due to a lack of a secure social network. In a recent Swedish study, homelessness was found to even increase mortality, particularly in connection with substance abuse.^8^

Despite the robustness of our findings, some limitations must be acknowledged. The retrospective nature of the study and the reliance on existing records might introduce biases related to data completeness and accuracy. Additionally, the exclusion of education level from the logistic regression model due to multicollinearity limits our ability to fully elucidate its impact relative to other variables.

## CONCLUSION

Our study highlights the statistically significant influence of socioeconomic factors on the hospitalization of patients with OIs. Lower income levels, single or non-married status, and rental living conditions are key predictors of hospitalization, reflecting broader issues of access to care, education, and living conditions. These findings emphasize the need for comprehensive public health strategies that address these socioeconomic disparities to improve oral health outcomes and reduce the burden of severe dental infections.

## ACKNOWLEDGEMENTS

Open access was funded by Helsinki University Library.

## REFERENCES
